# Supplementary material for: Are carbon emissions trading and green financial instruments synergistic? -Comprehensive quantitative research based on content analysis
Source: PLoS One. 2024 Mar 7;19(3):e0298601. doi: 10.1371/journal.pone.0298601 (PMC10919601; doi:10.1371/journal.pone.0298601)
Supplement: S1 Data — (ZIP) [file pone.0298601.s001.zip › Data/地级市Z_F工作报告.zip!/║╙─╧/Σ≡║╙╩╨/└┤╫╘╣1⁄2╓┌║┼í╛┬φ┐╦╩2╛▌═°í┐_2016.docx]

各位代表：

现在，我代表市人民政府，向大会报告工作，请予审议，并请各位政协委员和其他列席人士提出意见。

一、2015年及“十二五”工作回顾

2015年，面对严峻复杂的宏观经济形势，市政府在省委、省政府和市委的正确领导下，在市人大、市政协的监督支持下，和全市人民一道，以深化六项重大攻坚任务为统揽，凝神聚力，排难奋进，着力稳增长、调结构、促改革、惠民生，全市经济社会发展取得新成效，主要经济指标增速进入全省第一方阵。初步核算，全市生产总值992.9亿元，增长9%、居全省第6位;规模以上工业增加值641.4亿元，增长10.1%、居全省第4位;固定资产投资908.5亿元，增长17.5%、居全省第6位;社会消费品零售总额437.4亿元，增长13.2%、居全省第2位;一般公共预算收入68.3亿元，增长10.3%;城乡居民人均可支配收入17626元，增长9.9%;金融机构各项贷款余额501.7亿元，比年初增长19.7%，创历史最好水平。

——产业结构持续调整优化，经济发展水平实现新提升。食品、化工等主导产业提质增效，电子信息、生物医药等战略性新兴产业快速发展。全市规模以上工业主营业务收入2883亿元，增长10.2%;实现利润304亿元，增长9.2%;规模以上高新技术产业增加值142亿元，增长33.5%。第三产业发展加快，实现增加值261.5亿元，增长10.8%，这是2008年以来首次高于全省平均水平。电子商务蓬勃发展，全市各类电商企业超千家，4家企业被评为省级电子商务示范企业，创办了全省首家电商学院，东兴电商园成为全省电子商务示范基地。

——改革开放持续深入推进，发展动能不断增强。市、县政府机构改革顺利完成，公布了44个市政府部门和单位的权责清单。商事制度改革全面深化，我市颁发了全省第一张“三证合一”营业执照，新登记市场主体1.9万户，市场主体总量达到7.8万户，增长22.8%、居全省第1位。整合财政资金5亿元，设立了产业发展基金和企业信贷周转基金，着力解决中小企业融资难问题;争取债券资金24.1亿元，支持重大工程建设。民生银行、郑州银行等4家股份制金融机构入驻开业，未来五年将为我市投放资金500亿元。双建管桩、利通液压等5家优秀中小企业在“新三板”成功挂牌，实现境内资本市场融资新突破。事业单位分类改革持续深化，县级公立医院综合改革、交通运输行政执法体制改革、医药卫生体制改革、城乡居民养老保险制度改革、党政机关公务用车制度改革等得到落实。开放招商成果丰硕，全市新签约投资超亿元项目181个;实际利用外资8.5亿美元、实际利用省外资金208.9亿元，完成出口总额4.8亿美元;成功举办了第十三届中国(漯河)食品博览会;河南进口肉类指定口岸漯河查验区建成运营，智能物联港保税仓开工建设。155个市定重点项目完成投资389亿元，61个项目竣工投运;34个省定重点项目投资完成率达到122%，居全省前列。

——现代农业持续提升发展，农业基础地位更加巩固。全年粮食总产达到182.6万吨，实现了“十二连增”。临颍颍西、郾城西大坡30万亩示范方和舞阳泥河洼16万亩示范方成为全省样本、全国先进。新培育省级农业产业化集群4个、都市生态农业示范园区5个，新增生态规模养殖场22家，新增蔬菜种植面积 2.7万亩，新发展农民专业合作社170个、家庭农场80家。夏秋两季秸秆实现全面禁烧。全市土地流转面积达到135万亩，占家庭承包经营总面积的 58.5%。现代农业集约经营、规模经营水平明显提升，生态农业、生态畜牧业发展和生态林业建设走在全省前列，省政府两次在我市召开现场会。特别是全国春季农业生产现场会在我市召开，既充分展示了我市现代农业发展新水平，又为我市大力发展现代农业带来了新机遇，国家、省里在项目和资金投入上对我市给予大力支持。

——城乡建设持续加大力度，人居环境明显改善。市区150个城建项目完成投资231亿元。全面编制完成41项城市专项规划，我市被批准为全省第一个城市设计试点市。备受关注的黄山路、汉江路、嵩山南路等城市断头路成功打通，解放路沙河大桥主桥建成，太白山路沙河桥、澧河桥和道路贯通工程加快推进，龙江路升级改造全线开工建设，城市老居民区一万多户安全饮水问题全部解决，百座街头游园全部建成。城市基础设施和环卫设施进一步完善。“两城同创”工作成效明显，通过卫生城市国家级初审和文明城市国家级首次检查。县城、小城镇和新农村建设扎实推进。农村人居环境综合整治工作力度大、效果好，多数村庄卫生状况发生显著变化。

——民生质量持续改善提升，社会大局和谐稳定。省定10项民生工程、市定15件民生实事全面完成。就业再就业工作成效明显，社会保障体系更加完善。全市建成投用社区老年人日间照料中心15个、农村幸福院436个。市残疾人康复中心建成投用。新建110个留守儿童之家。全市新建、改扩建公办幼儿园81 所，建成了4所中小学;优化整合市区教育资源，市五高设立南校区初中部，市实验中学和市八中合并，市实验小学和西城区新建小学合并。市中心医院西城区分院已经建成，市二院、市六院病房大楼主体工程完工。全市所有乡镇卫生院都设立了大中医堂。文化、旅游、体育事业加快发展，成功举办了第三届许慎文化国际研讨会，我市成为全国首家“中国汉字文化名城”。生态环境建设持续加强，国土管理水平明显提升。统计、人防、气象、广播影视、新闻出版、外事侨务、民族宗教、史志档案等工作取得新成效;老龄、妇女、儿童、残疾人、慈善、红十字等事业得到新发展;精神文明建设成效显著，公民道德水平和文明程度实现新提升。国防教育、“双拥”工作继续加强，军民融合深入发展，驻漯解放军、武警消防官兵和民兵预备役人员为地方发展作出了重要贡献。食品药品监管得到强化，信访和安全生产形势持续平稳，平安漯河建设深入推进，应急处置能力明显提高，社会更加和谐进步。

一年来，我们自觉接受人大、政协监督和社会监督，认真办理人大代表议案、建议和政协提案，支持民主党派、工商联和无党派人士参政议政。依法治市和法治政府建设取得新进展。加大反腐倡廉建设力度，强化行政监察和审计监督。认真开展“三严三实”专题教育，深入落实中央八项规定和省委、市委贯彻意见，防止 “四风”反弹;健全权力运行制约和监管体系，开展懒政怠政为官不为专项治理，政风行风进一步好转。

各位代表!过去的一年，经济下行压力持续加大，实体经济面临重重困难。我们顶住压力、迎难而上，积极作为、敢于担当，科学应对、综合施策，及时出台稳增长保态势一揽子政策措施，实施稳增长扩投入保目标大决战，聚焦后进指标，狠抓关键环节，全市经济社会发展取得了高出预期的成绩，实现了“十二五”顺利收官，漯河发展站在了新的历史起点上!

五年来，在市委的坚强领导下，全市上下攻坚克难、奋力拼搏，在困境中闯关过坎，在压力下负重前行，破解了一道又一道难题，办成了一批又一批实事，经济社会发展每年都上新台阶，城乡面貌每年都有新变化。

这五年，综合经济实力显著提升。全市生产总值达到近千亿元，是“十一五”末的1.5倍;人均生产总值由“十一五”末的2.7万元提高到3.8万元;财政总收入突破120亿元，一般公共预算收入超过68亿元，分别比“十一五”末增加65亿元和42.2亿元，分别是“十一五”末的2.2倍和2.6倍;市级一般公共预算收入达到34.7亿元，比“十一五”末增加18.8亿元;县区一般公共预算收入平均超过6亿元，其中临颍县突破10亿元。三次产业结构进一步优化，第三产业增加值占全市生产总值的比重达到26.3%，比“十一五”末提高7个百分点。骨干企业群体快速壮大，全市税收超亿元企业10家、超千万元企业96家，分别比“十一五”末增加5家和61家;新增规模以上工业企业247家，新增年主营业务收入超10亿元工业企业45家;一大批中小企业茁壮成长，成为全市经济发展的生力军。产业集聚效应日益凸显，全市6个产业集聚区建成区面积达到60平方公里，实现主营业务收入2100亿元，从业人员超过20万人;商务中心区和特色商业区建设成效明显;千亿食品产业集群成为全省重点产业集群，电子信息、生物医药等战略性新兴产业成为新的经济增长板块;经济技术产业集聚区进入全省“十强”，临颍县产业集聚区升格为省级经济开发区;在每年的全省重点项目暨产业集聚区观摩讲评中，我市均取得好成绩。今年，省委、省政府将漯河与洛阳、新乡、许昌等市一同列为产业基础和配套条件较好的市，要求我市加快提质发展。这标志着我市在全省的地位和影响力又有了新的提升!

这五年，发展动力后劲显著增强。实际利用外资34亿美元、利用省外资金833亿元，均是“十一五”期间的3.1倍。争取上级转移性资金408亿元，其中地方政府债券34亿元。固定资产投资累计达到3400亿元，是“十一五”期间的2.6倍。取消、下放479项行政审批事项，财税金融、医药卫生、户籍制度等各领域改革全面深化，释放了更多改革红利。双汇集团成功并购美国史密斯菲尔德公司，成为全球最大的肉制品加工企业。新组建了市房投、农投等投融资公司，市城建投、发投、经投等公司投融资能力大幅提升。建成全省首家国家肉及肉制品检测重点实验室，双汇集团荣获省长质量奖，新培育国家级高新技术企业33 家，新增省级创新型企业15家，新建省级工程技术研究中心21个。专利申请量达到3690件，是“十一五”时期的2.5倍。交通基础设施建设实现新突破，沙颍河复航工程漯河至周口段即将建成、漯河至平顶山段已开工建设，漯河西高铁客运站通车运营，京港澳高速漯驻段改扩建工程、107国道和省道七蚁线、时南线、逍白线、常付线漯河段改建工程全面完成，全市公路通车总里程达到5249公里，公路密度居全省前列。

这五年，“三农”发展水平显著提高。全市累计投入和发放各类涉农资金64.2亿元。我市成功申创国家现代农业示范区、国家农业科技园区。新建高标准粮田106.5万亩。新增农民专业合作社2841个、家庭农场376家。蔬菜种植面积突破100万亩。新发展有效灌溉面积43.5万亩。全市综合农业机械化水平达到85.8%，高于全省平均水平8个百分点。南水北调配套工程建成投用，市区和临颍、舞阳两个县城已供上丹江口饮用水。中小河流域治理成效明显。新建、改造农村公路1263公里，新解决98.2万人的农村饮水安全问题，新建户用沼气池28.5万户。改造农村危房3.2万户。实现11.5万农村贫困人口稳定脱贫，完成了117个贫困村整村推进任务。成功创建6个国家级生态示范村、15个省级生态示范乡镇、139个农村人居环境示范村，广大乡村展示了新形象。

这五年，城镇面貌得到显著改观。全市城镇化率达到47.5%，比“十一五”末提高8.3个百分点。城市总体规划修编完成，中心城区控制性详细规划实现全覆盖。市区建成区由60平方公里扩大到76平方公里，常住人口由60万人增加到72万人。新建改造了124条道路、7座沙澧河大桥和一批供气、供热、供水、排水、环卫等设施，市区背街小巷改造全面完成、老旧小区全部整治、旱厕全面消除，全市污水日处理能力达到40万吨，垃圾无害化处理率达到98%以上。城市旧城区、棚户区和城中村改造稳步推进，累计完成房屋征收600万平方米、开工建设安置房260万平方米。城市绿化覆盖率39.7%、绿地率 33.7%、人均公共绿地面积11.6平方米。市城乡一体化示范区规划编制完成、机构组建到位，开发建设拉开序幕，各项工作全面展开。经济技术开发区食品产业特色凸显，名企名牌加快集聚，软硬环境不断优化，国家级开发区形象得到提升。西城区基础设施不断完善，项目建设力度加大，持续掀起开发建设高潮，初步展现了漯河新城区形象。沙澧河风景区成为全省唯一的国家体育公园，生态休闲沿河景观带已成为漯河的一张靓丽名片。临颍、舞阳两个县城建设成效明显，城区框架拉大、功能不断完善、形象大幅提升。乡镇建设力度加大，7个乡撤乡建镇，4个乡镇进入全省百强。

这五年，人民生活有了显著改善。全市各级财政累计用于民生支出461亿元，占一般公共预算支出的73.6%，其中2015年达到124.9亿元。城镇新增就业29万人，农村劳动力转移就业新增18.5万人。社会保险基本实现全覆盖，企业退休人员养老金、城镇职工基本医疗保险最高支付限额全面提高。新农合保障能力明显增强，农民参合率达到99%，共补偿参合农民27亿元。城市低保标准每月450元，农村低保标准每月220元，农村五保集中供养标准每年 3800元，和“十一五”末相比，分别提高157%、120%和70%。社会养老服务体系、社会救助体系和社会福利体系进一步完善。新建各类保障性住房 7.8万套，为7.3万户低收入家庭圆了安居梦;发放住房公积金贷款32亿元，帮助4.6万户职工改善了居住条件。新建214所幼儿园、12所中小学，4 个县区通过国家义务教育均衡发展评估认定，普通高考本科上线人数连年保持万人以上，职业教育活力不断增强，高等教育质量持续提升。城乡医疗卫生服务体系进一步完善，市、县区医院病房楼和急救中心，乡镇卫生院和村卫生室改扩建等一大批项目建成投用。许慎文化、贾湖文化、舞阳农民画、“幸福漯河”系列群众文化活动等品牌进一步打响，举办了100多项省级以上高水平体育赛事，新建近700个农村综合性文化体育活动中心，城市展示馆和市图书馆、博物馆、群艺馆即将建成投用，“市民之家”和青少年示范性综合实践基地项目已开工建设。经过五年的不懈努力，我们正在干一批提升漯河形象、完善城市功能的重大工程，干成了一批群众期盼多年的事情，群众的获得感明显增强。

各位代表!“十二五”时期是漯河发展历程中不平凡的五年。五年来，全市人民苦拼实干，经济社会发展实现了新突破、取得了新业绩、增创了新优势，漯河发展的基础更加稳固、空间更加广阔、动力更加强劲。这些成绩的取得，是省委、省政府和市委正确领导的结果，是市人大、市政协监督支持的结果，是全市人民齐心协力、艰苦奋斗的结果。在此，我代表市人民政府，向工作在各条战线的工人、农民、知识分子、干部，向驻漯部队、武警消防官兵、公安民警以及各界人士，致以崇高的敬意!向所有关心支持漯河发展的同志们、朋友们，表示衷心的感谢!

回顾“十二五”发展历程，成绩来之不易，奋斗充满艰辛，经验弥足珍贵。我们感触颇多、深有体会。

一是必须咬定发展不放松。无论外部环境如何变化，始终牢牢把握发展第一要务，突出重点、攻克难点、打造亮点，坚持做强工业壮实力、做大三产补短板、做优农业强基础，把开放招商作为“一号工程”、把项目建设作为重要抓手、把产业转型升级作为主攻方向，强力实施工业1351工程，出台了促进战略性新兴产业、现代服务业、房地产业发展等一系列政策措施，多措并举，持续发力。特别是在经济下行压力加大的严峻形势下，深入研判宏观形势，加强经济运行监测，不断深化企业服务，全力稳增长、保态势，持续调结构、促转型，在克难攻坚中推动漯河加快发展。

二是必须敢于担当强突破。面对各种热点难点问题，敢抓敢管、敢于碰硬，豁得出去、顶得上来。积极搭建投融资平台，创新投融资方式，整合各类财政性资金，支持城市建设和城乡基础设施建设，支持现代农业和高新技术产业发展，着力破解影响发展的资金瓶颈制约。实施城市建成区高污染、高耗能企业“退二进三”，调整优化产业结构，进一步改善城市环境。大胆创新思路，完善城市“两改”政策，有效解决拆迁难题。强力推进城市公交改革，优化城市公交线路，配置新型节能环保车辆，树立城市公交新形象。在全省率先回购商品房作为公租房、出台购房补贴政策、推行“公转商”模式，促进住房消费，加快商品房库存消化，推动房地产业健康发展。健全完善规划管理体制和城市管理体制，提升城市精细化管理水平。理顺市城乡一体化示范区、西城区的管理体制和财政体制，为两个区的大开发、大建设积极创造有利条件。

三是必须尽心竭力惠民生。坚持把改善民生作为一切工作的出发点和落脚点，做决策、办事情，充分听取群众意见，充分考虑群众利益，在任何时候都把改善民生放在心上、抓在手上、落实在行动上，从解决群众最关心的问题抓起，从办好群众最希望的事情做起，在财政收支矛盾加剧的情况下，持续加大民生投入，每年都坚持办好一批民生实事，尽最大努力改善群众生活、提升民生质量。

四是必须上下同心聚合力。面对严峻复杂的宏观环境，面对日益激烈的区域竞争，全市上下紧紧围绕发展目标和工作重点，增强压力、增添动力、激发活力，持续转变作风、持续简政放权、持续高效服务，各县区竞相发展，各部门顾全大局，各级领导率先垂范，广大干群合力攻坚，形成了心齐、风正、劲足的良好局面，打造了一批工作亮点。我市被确定为全国质量强市示范城市创建城市、全国养老服务业综合改革试点市、全省住房保障创新试点市、全省行政审批标准化试点市，荣获全国双拥模范城、全国首批法治城市创建先进市等称号。特别是去年国务院决定在我市召开全国春季农业生产现场会，筹备工作时间紧、任务重、要求高，全市上下快速行动、周密安排、精心筹备，大会取得圆满成功，我市的城乡面貌、城市管理、现代农业、接待服务等受到国家领导人和与会人员的高度评价，漯河的知名度和影响力得到大幅提升!

五是必须工作抓底重落实。对重点任务，明确分管领导、责任单位、完成时限，盯紧抓牢每季、每月、每个节点，强化过程管理，强力督查问效，定期观摩评比，严格考核奖惩，上级政策和市委、市政府决策部署得到全面落实。在去年国务院开展的大督查中，我市以优异成绩名列全国受到表扬的20个地级市首位，今年将享受国家资金支持等五个方面的激励政策，省政府也将对我市予以表扬和奖励。年度综合性经济工作受到国务院的表彰，在我市尚属首次，这是全市上下辛勤努力、勇于担当、务实重干、奋发作为的丰硕成果!是肯定漯河、宣传漯河、鼓舞漯河，激发全市人民干事创业热情的正能量!

各位代表!我们也清醒地认识到，前进道路上还面临不少困难和问题，政府工作与人民的期待还有一定差距。我市经济总量还不大，实力还不强，家底还不厚实;产业层次不够高，战略性新兴产业规模不大，转型升级任务艰巨;重污染雾霾天气多发;城市基础设施仍有欠缺、功能不够完善;改善民生任重道远;政府职能转变还不到位，一些部门和工作人员还存在着懒政怠政、为官不为等问题。对此，我们一定高度重视，直面问题，采取有力措施，认真加以解决，决不辜负全市人民的重托和期望!二、“十三五”时期的奋斗目标和主要任务

“十三五”时期是全面建成小康社会的决胜阶段，也是我市爬坡过坎、攻坚转型、增强实力的关键时期。必须准确把握经济发展新常态带来的趋势性变化和我市所处的历史方位，以滚石上山的精神和勇气，奋力开拓发展新境界，使漯河在未来发展中勇立时代潮头!

根据市委“十三五”规划建议，市政府编制的《漯河市国民经济和社会发展“十三五”规划纲要(草案)》及说明已印发会议，请一并审议。

“十三五”时期全市经济社会发展的指导思想是：高举中国特色社会主义伟大旗帜，全面贯彻党的十八大和十八届三中、四中、五中全会精神，以马克思列宁主义、毛泽东思想、邓小平理论、“三个代表”重要思想、科学发展观为指导，深入贯彻习近平总书记系列重要讲话精神，坚持“四个全面”战略布局，坚持发展是第一要务，树立创新、协调、绿色、开放、共享的发展理念，以提高发展质量和效益为中心，以深化六项重大攻坚任务为统揽，着力四化同步科学发展，着力壮大总量增强实力，着力优化结构转型升级，着力深化改革强化动力，着力改善民生促进和谐，统筹推进经济建设、政治建设、文化建设、社会建设、生态文明建设和党的建设，推动富民强市加快发展，确保如期全面建成小康社会，努力在新一轮改革发展大潮中继续走在全省前列。

“十三五”时期主要预期目标是：主要经济指标年均增速高于全国、全省平均水平，生产总值、城乡居民人均收入比2010年翻一番以上，综合经济实力跻身全省第一方阵;公共服务体系更加健全，社会保障体系日益完善，人民生活水平全面提高;生态环境质量明显改善，天蓝、地绿、水净的美丽漯河全面展现;城市品质、全民素质和社会文明程度持续提升，努力成功创建全国文明城市、国家卫生城市;各方面体制机制更加完善，治理体系和治理能力现代化取得重大进展;到 2020年全面建成小康社会，加快现代化建设步伐，打造一批在全省乃至全国的特色亮点，使漯河在全省大格局中的地位作用和影响力显著提升。

实现“十三五”目标，我们必须以新理念引领新发展，聚焦五大发展理念、聚焦全面建成小康社会、聚焦“一区两城一中心”、聚焦六项重大攻坚任务，推动实现更高质量、更有效率、更加公平、更可持续发展。未来五年，重点抓好五大任务。

(一)坚持创新发展，实现产业转型升级新突破。深入实施创新驱动战略，全面提升发展质量和效益。着力抓好供给侧结构性改革，提升科技创新能力，推动产业向中高端迈进，加快建设最具竞争力的中国食品名城、全省重要的电子信息产业基地和生物医药产业基地、区域性现代商贸物流中心、国家现代农业示范区。实施 “互联网+”行动计划，用“互联网+”思维创新创造，拓展更大发展空间。

(二)坚持协调发展，构建城乡统筹发展新格局。加快中心城市、县城、小城镇和新农村建设，持续推进以人为核心的新型城镇化。彰显城市特色，丰富城市内涵，完善城市功能。推进城市组团发展，建设市区与临颍、舞阳的快速通道。抓好县城建设扩容提质，培育一批特色小城镇，打造一批美丽宜居乡村。健全城乡发展一体化体制机制，逐步实现城乡基本公共服务均等化、资源配置合理化、产业发展融合化。

(三)坚持绿色发展，建设生态宜居美丽新家园。依托沙澧河的持续开发，大力加强生态水系建设，打造中原水城。以创建国家生态园林城市为载体，打造生态绿城。推动低碳循环发展，积极发展新能源产业。深入实施大气、水、土壤污染防治行动计划，推进工业污染源全面达标排放，实现城镇生活污水垃圾处理设施全覆盖。

(四)坚持开放发展，全面激发经济发展新活力。大力实施开放带动战略，努力打造中原开放高地。主动融入国家“一带一路”建设，加强与郑州航空港经济综合实验区深度对接和战略合作。围绕主导产业，瞄准重点区域，实施精准招商。坚持办好中国(漯河)食品博览会，提高专业化、市场化水平。加快开放平台建设，坚持引进来与走出去并重，全面提升开放型经济发展水平。

(五)坚持共享发展，努力实现民生质量新提升。持续加大民生投入，提高公共服务共建能力和共享水平。坚决打赢脱贫攻坚战，2017年，舞阳县摘掉省级扶贫县帽子，其他贫困人口实现精准脱贫。完善就业服务和社会保障体系，推动大众创业万众创新。大力发展教育事业，努力办好人民满意的教育。提高城乡医疗卫生服务水平，推进健康城市建设。创新社会治理方式，切实增强人民群众的幸福感和满意度。

各位代表!宏伟蓝图已经绘就，美好前景催人奋进。只要我们万众一心、真抓实干、奋力前行，就一定能够实现全面建成小康社会目标，就一定能够再创漯河发展新的辉煌!三、2016年工作安排

今年是实施“十三五”规划、全面建成小康社会决胜阶段的开局之年，是推进结构性改革的攻坚之年，也是漯河建省辖市三十周年，做好今年的政府工作十分重要。我们必须认清形势、把握大势，直面挑战、坚定信心，以更大的气魄、更强的力度、更有效的举措，努力实现“十三五”良好开局。

今年的主要预期目标是：生产总值增长8%以上，规模以上工业增加值增长9.5%左右，第三产业增加值增长9%，一般公共预算收入增长8%，固定资产投资增长16%，社会消费品零售总额增长13%，城乡居民人均可支配收入增长9%左右，居民消费价格涨幅控制在3%左右。节能减排、环境质量改善等约束性指标完成省下达任务。

围绕上述目标，今年要着力抓好八项重点工作。

(一)突出转型升级，着力提高经济发展质量和效益。积极落实国家供给侧改革政策措施，为稳定增长、提质发展提供强大动力。

做大做强工业。大力发展战略性新兴产业。重点培育电子信息、生物医药、装备制造等新兴产业，加快东兴电商产业园、吉顺电子工业园、曙光健士医疗器械、漯河大健康产业园、舞阳医药化工园、临颍智能装备产业园、经济技术开发区装备产业园等项目建设。加快传统产业优化升级。依托双汇、旺旺、嘉吉、可口可乐等知名品牌，提升发展食品产业。开工建设上好佳休闲食品产业园等项目，加快旺仔牛奶、雅客休闲食品工业园、三剑客乳制品工业园、天冠嘉吉产业园等项目建设。今年，全市规模以上食品工业主营业务收入力争达到1800亿元。支持盐化工业精深开发，加快技术创新，提升产业竞争力。引导造纸、冶金、纺织、制革制鞋等传统产业加快技术改造，降低企业成本，增强发展活力。壮大骨干企业群体。全面完成工业1351工程目标任务，培育年销售收入超5亿元、税收超千万元的龙头企业30家，新增规模以上工业企业50家。加快企业上市步伐，力争新增上市和挂牌企业10家以上。大力扶持小微企业，推动企业创新管理、提质增效。加大危困企业帮扶力度。对产品有市场、发展有潜力而一时存在困难的企业，政府要积极施策帮助。

强化载体建设。产业集聚区要提升集群优势，打造千亿食品产业集群和电子信息、生物医药、盐化工、装备制造4个百亿级特色产业集群;提升基础设施，加快新扩区域基础设施建设;提升服务功能，推进产业集聚区信息基础设施建设，加快完善区内行政服务中心功能;提升机制活力，力争上半年完成区镇(办)管理套合。在服务业“两区”建设上，市商务中心区重点提升区域综合服务功能，推动金融服务、设计研发、企业总部、区域结算中心等加快集聚;各县区特色商业区要突出特色化、差异化发展，着力抓好品牌集聚、业态创新、管理服务，尽快打造特色和亮点。

加快服务业发展。大力发展现代物流和商贸流通业，持续打造食品冷链、粮食及农产品、家电、汽车及零配件等物流品牌，着力抓好昌建国际广场、荣昌商业广场、开源新城商业综合体、中王奥特莱斯综合体、闽豫国际建材港、沙河港物流园区等一批重点项目建设。积极发展电子商务等新兴服务业，提升发展文化旅游等高成长性服务业，推广应用大数据、云计算、物联网、互联网等新技术，促进服务业提速度、扩规模、上水平。

推动房地产业健康发展。认真落实去年市政府出台的促进房地产业健康发展的有关政策措施，以去库存、促消费为重点，建立购租并举的住房制度，鼓励临颍、舞阳两县出台吸引农民进城购房的优惠政策，进一步用足用好住房公积金，将有稳定劳动关系的常住人口纳入公积金覆盖范围，扩大“公转商”规模，畅通商品房和保障房转换通道，促进商品房销售。努力提高棚改货币化安置率，其他征地拆迁项目也要大力推进货币化安置。积极发展住房租赁市场，推动住房租赁规模化、企业化。鼓励企业利用存量房发展养老、创业等地产，开展多元化经营。

(二)突出项目支撑，着力扩大有效投资。坚持投资拉动不动摇，优化投资结构，提高投资效益。实施千亿投资计划，重点安排建设项目390个，年度计划完成投资700亿元以上，带动全社会固定资产投资突破1000亿元。

狠抓重点项目建设。今年，安排市定重点项目155个，年度计划完成投资400亿元以上，计划新开工重点项目80个以上、竣工60个以上。完善项目推进机制，强化项目建设和管理，推动项目早开工、早投产、早见效。

破解投资瓶颈制约。积极推进社会资本通过PPP模式，参与城镇基础设施建设。认真研究国家政策性资金投向和专项建设基金政策，抓住我市受到国务院表扬奖励的机遇，积极争取上级政策性项目资金。发挥好产业基金的作用，对重点项目进行扶持。落实重点项目用地分类保障制度，确保重点建设项目用地指标专项使用、落到实处。

加强项目谋划储备。围绕壮大支柱产业、培育新兴产业、完善基础设施、加快民生改善等重点工作，结合“十三五”规划编制，谋划储备一批重大项目，增强投资后续支撑能力。

(三)突出开放招商，着力增强发展活力。适应开放新形势，顺应招商新趋势，持续加大招商力度，全面提升开放水平。

提高招商实效。创新招商方式，深化产业招商，加强驻地招商，提升园区招商，推进以商招商，提高招商的精准性，形成引进一个、带来一批的联动效应。精心组织系列有针对性的主题招商活动，积极参加国家、省举办的重大经贸活动，认真办好第十四届中国(漯河)食品博览会，持续掀起招商高潮。完善招商责任推进机制，深入开展签约项目专项督查，进一步提高签约项目合同履约率、资金到位率和项目开工率。加强落地项目后续服务工作，确保项目落得下、建得快、发展好。

拓宽开放领域。进一步扩大现代服务业、现代农业、基础设施和社会事业等领域开放，力争引进一批合作项目。全面清理限制非公有制经济发展的不合理规定，大力引进外来资本和民间资本。抢抓“一带一路”战略机遇，找准对接点，深化战略合作，大力培育出口龙头企业，支持优势企业和产品走出去。

优化开放环境。充分发挥河南进口肉类指定口岸漯河查验区的作用，完善肉类口岸功能。力争智能物联港保税仓早日建成投运。积极推进漯河海关申报工作，启动电子口岸建设。充分发挥产业集聚区的招商平台作用，完善功能，精心包装，强化推介，使其成为全市开放招商的重要载体。

(四)突出固本强基，着力发展现代农业。以建设国家现代农业示范区为统领，坚持集约高效绿色可持续发展，促进农业增效、农民增收、农村繁荣。

提高粮食生产能力。落实藏粮于地、藏粮于技战略，实行最严格耕地保护制度。持续推进高标准粮田建设工程，新建高标准粮田10万亩。实施耕地质量保护与提升行动，大力推广土地深松等技术，加强农田水利建设，加快农业科技创新及成果转化，加大农业技术推广力度，巩固提高粮食综合生产能力。

调整优化农业结构。大力发展特色农业、高效农业、绿色农业和品牌农业，深入实施现代农业产业化集群培育工程、生态畜牧养殖工程和都市生态农业发展工程。加快粮经饲统筹、农林牧结合、种养加一体、一二三产业融合发展，推动农业规模化经营、标准化生产、品牌化营销。

提升综合服务水平。完成农村土地承包经营权确权登记颁证工作，基本完成农村集体建设用地使用权确权登记发证工作。落实农业“三项补贴”改革，发展种养大户、家庭农场等新型农业经营主体，培育新型职业农民，健全农业公共服务和社会化服务体系。完善临颍县“土地银行”的经验做法，适时在全市推广。

(五)突出城乡统筹，着力推进新型城镇化。城镇化率偏低是我市经济社会发展的一个短板，要重点抓好新型城镇化工作，加快提高户籍人口城镇化率，切实强化新型城镇化带动作用，促进城乡发展一体、区域发展协同。

加快中心城市建设。今年，安排城建项目174个，力争完成投资236亿元。实施一批路桥工程，城区北部贯穿东西的龙江路改造提升工程要全面建成，城区西部连三区、跨两河的太白山路要全线贯通，解放路路桥工程竣工通车，中山路上跨立交桥建成投用，打通交通北路、泰山北路等一批断头路，改造人民路澧河桥，加快建设漯河沙河航运港、牡丹江路路桥工程、沙澧连通工程、示范区段沙河景观工程、沙颍河复航城区段改造工程。推进龙江生态城、五里庙城市综合体等节点街区建设，抓好园林绿化建设、城区次干道整修、市场改造等惠民工程。加快建设沙北热电厂。稳步推进城市旧城区、棚户区和城中村改造，完成房屋征拆面积110 万平方米、建设安置房6000套。市城乡一体化示范区、经济技术开发区、西城区要立足各自实际，结合功能定位，切实抓好重点工程建设。深入推进“两城同创”，力争早日实现创建目标。

抓好县城、小城镇和新农村建设。加大临颍、舞阳两个县城建设管理力度，加快颍北新区、舞北新区建设，使县城发展成为吸纳农民进城的主阵地。重点抓好市区周边的孟庙、姬石等10个卫星镇建设，实现与中心城区融合发展;推进繁城、北舞渡等10个中心镇的镇容镇貌综合整治，提升综合承载能力。分类推进新农村建设，着力抓好农村公路、安全饮水等基础设施建设，深入开展农村人居环境综合整治，积极探索农村垃圾、污水集中治理措施，建立村庄保洁长效机制，加快建设美丽宜居乡村。

(六)突出改革创新，着力释放发展动能。围绕解决突出问题，充分调动各方面改革创新热情，增强发展内生动能。

深化重点领域改革。深化政府职能转变，开展省定行政审批标准化试点，全面落实权责清单制度。稳妥推进事业单位改革。深化商事制度改革，扩大“三证合一”改革成果。深化投融资体制改革，建立多元化融资平台。深化金融体制改革，加快组建漯河农商行，积极引进新的金融机构入驻我市。深化财税体制改革，清理、整合、规范专项转移支付和专项资金项目，推进营改增扩围、消费税改革和资源税费改革。深入推进教育、文化、社保等领域改革，确保取得新成效。

深化科技创新。强化企业技术创新主体地位，推动创新成果向企业集中、创新政策向企业倾斜、创新人才向企业流动。加快企业研发中心建设，新培育国家级高新技术企业5家以上，新建省级工程技术研究中心3家以上。支持产业集聚区、服务业“两区”和专业园区建设研发创新平台。加强创新队伍建设，引进、培养一批高端人才，攻克一批关键技术。发挥“互联网+”的创新引擎作用，加快推动产业转型升级。

(七)突出环境保护，着力加强生态建设。实施最严格的环境保护制度，持续实施蓝天、碧水和乡村清洁工程，大力推进绿色发展、循环发展、低碳发展，加快建设美丽漯河。

强化污染防治。坚持源头严防、过程严管、后果严惩，抓好大气污染治理，遏制雾霾天气高发;加强水污染防治，深化重点流域河流污染治理，确保7条省控河流断面水质达标。加强污染物排放总量控制。加快建设沙北污水处理厂二期工程，开工建设马沟污水处理厂。开展环保综合督查和重点领域专项执法检查，严厉打击环境违法行为。

推行资源节约循环利用。推进生产、流通、消费各环节循环经济发展。全面实行城乡生活垃圾、餐厨垃圾、医疗垃圾的集中处理。培育低碳产业，发展太阳能光伏、生物质能等新能源。倡导低碳消费方式。

完善生态绿化系统。加强农田林网建设，持续抓好村镇绿化。实施生态廊道提升工程、城市道路绿化景观提升工程、城市绿化细胞工程，推进绿色生态城区建设，提升城市绿化水平。

(八)突出民生改善，着力增进人民群众福祉。以实施省定民生工程、市定民生实事为重要抓手，切实保障和改善民生，加快健全基本公共服务，维护社会和谐稳定。

打好脱贫攻坚战。贯彻精准扶贫、精准脱贫基本方略，坚持转移贫困劳动力就业、扶持农村特色产业、加强知识技能培训、改善农村生产生活条件、实行社会政策兜底、实施扶危救困“六措并举”，促进贫困地区群众精准脱贫。全年完成贫困村整村推进40个，实现3.4万农村贫困人口稳定脱贫。

抓好就业创业和居民增收。积极推进创业型城市创建，落实好全民技能振兴工程，千方百计扩大就业。全年城镇新增就业3.5万人，农村劳动力转移就业新增 2.5万人。健全企业职工工资正常增长和支付保障机制;认真落实上级关于提高机关事业单位人员工资政策，落实好县以下机关公务员职务与职级并行制度和乡镇工作补贴;持续增加城镇中低收入者收入，多渠道增加农民经营性、工资性和财产性收入。

切实加强社会保障工作。全面实施全民参保登记计划。深化机关事业单位养老保险制度改革。提高企业退休人员基本养老金，提高职工医保、城镇居民医保政策范围内住院费用支付比例，提高大病保险人均筹资水平和支付比例。统筹推进城乡社会救助体系建设，完善最低生活保障、特困人员供养、残疾人等兜底性制度，深入开展社会救助，全面落实城乡低保发放、大病医疗救助、临时困难救助、农村五保供养等政策。抓好全国养老服务业综合改革试点工作。加快建设市养老产业综合服务园，开工建设昌建公司西城区养老产业园，抓好社区老年人日间照料中心、农村幸福院建设。积极推进医疗卫生与养老服务相结合。发展红十字事业，鼓励慈善捐赠和志愿服务。健全农村留守儿童、妇女、老人关爱服务体系。

加快发展社会事业。优先发展教育。实施第二期学前教育三年行动计划;所有县区全部通过国家义务教育均衡发展评估认定，市区建成4所中小学，高中教育质量继续保持全省领先;职业教育特色更加凸显，加快推进漯河技师学院新校区、漯河食品职业学院扩建、市第一中专搬迁等项目建设;支持漯河医专、漯河职院、漯河食品职业学院申办本科。鼓励、支持社会力量办学。加强文化建设。积极践行社会主义核心价值观，广泛宣传“崇文尚德、务本图强”新的漯河精神，开展丰富多彩的群众性精神文明创建活动，大力弘扬传统文化，持续扩大许慎文化、贾湖文化、舞阳农民画和“幸福漯河”系列群众文化活动的影响力，不断丰富汉字文化名城内涵，深入推进农村综合性文化体育活动中心建设，加快文化旅游景区提档升级步伐，加强公共体育设施建设，建成城市展示馆和市图书馆、群艺馆、博物馆，抓紧推进“市民之家”、青少年示范性综合实践基地项目建设。完善健康服务体系。深化医药卫生体制改革，抓好城市公立医院改革试点和县级公立医院综合改革。健全农村医疗卫生服务体系和重大疾病防控体系。市中心医院西城区分院，市二院、市六院病房楼，临颍县、舞阳县、郾城区中医院病房楼，源汇区、召陵区疾病预防控制中心实验楼建成投用，完成乡镇卫生院、村卫生室建设项目。全面落实放开“两孩”政策。同时，做好统计、气象、广播电视、新闻出版、外事侨务、民族宗教、史志档案等工作。支持工会、共青团、妇联等人民团体发挥桥梁纽带作用。加强国防教育和国防后备力量建设，支持驻漯解放军、武警部队、预备役部队、民兵和人民防空建设。深入开展双拥共建，积极创建新一届全国双拥模范城，进一步巩固和发展军政军民团结。

创新社会治理。健全社会治安立体防控体系，完善反恐怖工作和打击犯罪机制，强化流动人口和特殊人群服务管理。做好信访维稳工作，更加注重依法化解社会矛盾。强化食品药品安全监管。严格落实安全生产责任制，深化重点行业领域安全生产专项整治，坚决防止重特大事故发生。防范和化解各类风险隐患，最大限度地维护群众利益、维护经济发展、维护社会稳定。四、全面加强政府自身建设

新形势、新任务对政府工作提出了新要求，我们必须自觉践行“三严三实”、持续加强自身建设，付出艰辛努力、务求更大作为，为人民造福、让人民满意。

重法治，讲规范，严格依法行政。带头尊法学法守法用法，把政府工作全面纳入法治轨道，切实做到法定职责必须为、法无授权不可为。自觉接受人大及其常委会法律监督、工作监督和政协民主监督，认真办理人大代表议案、建议和政协提案，广泛听取民主党派、工商联、无党派人士和人民团体意见。完善重大决策合法性审查机制，让政府的每一项决策都经得起法治的考量、体现出民意的力量。提高行政执法水平，完善行政复议制度，全面启动“七五”普法，做好法律援助工作。加强行政监察、审计监督和政府内部层级监督，主动接受司法监督、社会监督和舆论监督，深入推进政务公开，确保权力在阳光下运行。

强宗旨，优服务，全心全意为民。始终把人民的期盼放在首要位置，密切联系群众，倾听群众呼声，回应群众关切，把更多的资源投入到为民服务上，真心实意为群众办实事、做好事、解难事。进一步简政放权，优化审批流程，简化办事程序，缩短审批时限，畅通为民服务“最后一公里”。切实改进服务方式，强化服务意识，提升服务水平。持续深化企业服务活动，集中整治企业周边环境和项目建设环境，深入开展“百姓问政”等活动，认真做好市长热线和政风行风热线办理工作，着力打造更加优良的发展环境。

转作风，提效能，狠抓工作落实。坚持求真务实，脚踏实地，工作抓底，埋头苦干。坚持问题导向，深入开展调查研究，到基层一线去发现问题、研究问题、解决问题。精简会议文件，改进会风文风。扎实开展政府作风建设年活动，狠治庸政懒政怠政，着力解决慢作为、不作为、乱作为等问题，切实营造肯干事、能干事、干成事的浓厚氛围。加大督查力度，创新督查方式，切实做到事前建账、事中查账、事后算账，着力整治重布置、轻落实、推诿扯皮、敷衍塞责等现象，确保每一项工作善始善终、善做善成。

勇创新，敢担当，全力以赴攻坚。面对新问题、新矛盾，勇于创新、善于创新，坚决破除制约发展的思想障碍，积极探索新常态下做好工作的方法和经验，创新发展路径，完善政策举措，着力在优化结构、补齐短板、深化改革、增强动力、化解矛盾、维护稳定上取得突破性进展。强化担当意识，敢于在加快发展中担重任、在深化改革中涉险滩、在重点工作推进中啃硬骨头，遇到困难不退缩、面对矛盾不回避，始终保持干事创业的热情、动真碰硬的豪情、奋发有为的激情，不断创造经得起实践、群众和历史检验的业绩。

严要求，守纪律，清正廉洁从政。坚持把纪律和规矩挺在前面，严格遵守《中国共产党廉洁自律准则》和《中国共产党纪律处分条例》。狠抓反腐倡廉，认真落实主体责任和“一岗双责”。加大违纪违法案件查办力度，强化巡察监督，严肃查处发生在群众身边的腐败问题。巩固党的群众路线教育实践活动和“三严三实”专题教育成果，持续推进正风肃纪。加强对重点领域、关键环节、重要岗位的监督制约，坚持用制度管权、管事、管人。牢固树立过紧日子思想，从严从紧控制“三公”经费，切实把更多财力用于发展经济、保障民生。加强公务员教育、管理和监督，做到干净干事、清白做人，树立为民务实清廉新形象。

各位代表!未来五年的宏伟蓝图鼓舞着我们，全市人民的热切期盼激励着我们，漯河发展的美好前景召唤着我们。让我们在市委的坚强领导下，继往开来，团结拼搏，务实重干，奋发作为，共同谱写漯河发展更加辉煌的新篇章!
